# Temporal-spatial distribution characteristics of leprosy: A new challenge for leprosy prevention and control in Zhejiang, China

**DOI:** 10.1371/journal.pntd.0008956

**Published:** 2021-01-07

**Authors:** Limei Wu, Yunliang Shen, Qiang Yao, Xudong Sang, Lijuan Fei, Wenming Kong, Ye Huang, Yanmin Wang, Fanrong Zeng, Na Du

**Affiliations:** Zhejiang Provincial Institute for Dermatoses Prevention and Cure, Deqing county, Huzhou City, Zhejiang Province, China; Lowell General Hospital, UNITED STATES

## Abstract

**Background:**

After the elimination of leprosy in 1995, there were 10–30 newly detected leprosy cases every year in Zhejiang Province, and the epidemiological characteristics of the newly detected leprosy cases have changed. While most of the newly detected cases came from other provinces in China, not Zhejiang, it brought a new challenge for leprosy prevention and control in post- elimination era in Zhejiang, China. This study was aimed to understand the temporal-spatial distribution characteristics of newly detected leprosy cases, and provide the scientific rationales for the development of leprosy control strategy.

**Methods:**

Data on the demographic of Zhejiang Province from 2011 to 2019 were obtained from the China Information System for Disease Control and Prevention, and the epidemiological data on leprosy cases newly detected in Zhejiang Province from 2011 to 2019 were obtained from the LEPROSY MANAGEMANT INFORMATION SYSTEM IN CHINA (LEPMIS), and temporal-spatial distributions were described. The geographic information system software—ArcGIS 10.4 was used to draw the statistical maps, and Geoda 1.14.0 was used for local spatial autocorrelation analysis (local Getis coefficient method). Ridley-Jopling classification was used to classify the clinical types into I, TT, BT, BB, BL or LL. Two-group classification system developed by the World Health Organization (WHO) was used and cases were classified into multibacillary (MB) type or paucibacillary (PB) type.

**Results:**

A total of 167 leprosy cases were reported in Zhejiang Province during 2011–2019, including 107 cases in males and 60 in females. The mean age at diagnosis was 37.99±14.81 years, and 94.01% of the cases were detected through the examination at skin-clinics. The number of workers, MB cases, G2D cases were 81 (48.50%), 159 (94.01%), 24 (14.37%) respectively, and the rate of early detection increased from 45.16% in 2011 to 90.91% in 2019. Leprosy cases were reported in all the prefectures of Zhejiang except Zhoushan City. The cases in local population accounted for 23.35% (39 cases), and the cases in floating population (especially coming from high epidemic provinces in China) accounted for 76.65% (128 cases). The annual number of newly detected cases showed a decreasing trend, from 31 cases in 2011 to 11 in 2019. Time of the floating population living in Zhejiang Province ranged from several months to more than 10 years. The annual proportion of new cases with G2D declined from 22.58% in 2011 to 9.09% in 2019. The results of local indicators of autocorrelation (LISA) analysis showed that the high-high areas were mainly concentrated in the middle and northeast of Zhejiang Province, while the low-low areas were in the east and southwest.

**Conclusion:**

A few scattered cases still can be seen in post-elimination era, and there was a spatial clustering of the newly detected leprosy cases in Zhejiang Province. Most of the cases in Zhejiang Province were from other high epidemic provinces in China, which brought a new challenge for leprosy control and prevention in post- elimination era in Zhejiang, and it is also necessary to strengthen the early detection and standard management of the leprosy cases in floating population in Zhejiang.

## Author summary

After elimination, most of the leprosy cases were detected passively, and the epidemiological characteristics of the newly detected leprosy cases had been changed. The proportion of floating population from other provinces in China increased year by year. In 2011, Health Commission of Zhejiang Province developed the planning of reducing harm of leprosy (2011–2020) and implemented leprosy symptom monitoring system in order to strengthen the early detection of leprosy cases. 167 newly detected cases during 2011–2019 were analyzed, whose factors were associated with household registration of other provinces, G2D and delay in diagnosis were explored. Annual proportion of household registration of other provinces showed an increasing trend, from 77.42% in 2011 to 81.82% in 2019, while G2D and delay in diagnosis among newly detected cases showed declining trends, from 22.58%, 40 months in 2011 to 9.09%, 11 months in 2019 respectively. In late phase of leprosy control, some kinds of leprosy control program are still necessary. Control and prevention strategy of floating population based on government commitments would be beneficial to improve early detection of leprosy cases.

## Introduction

Leprosy is an age-old, chronic infectious disease caused by Mycobacterium leprae, an acid-fast, rod-shaped bacillus. This disease is usually endemic in tropical countries, especially in developing countries [1]. The bacterium has a long incubation period (on average five years or longer). The disease affects nerve endings and destroys the body’s ability to feel pain and injury. If not treated in the early stages of infection, leprosy, a curable disease, can result in permanent damage to peripheral nerves and may lead to disabilities, and leprosy is considered as a severe public health issue [2].

Since the successful introduction of multidrug therapy (MDT) recommended by WHO in 1981, and the implementation of " Global Leprosy Strategy 2016–2020: Accelerating towards a leprosy-free world " In 2016, the global prevalence rate of leprosy declined dramatically. A general decrease was seen in the number of new cases detected globally, from 244,796 in 2009 to 208,619 in 2018 [3]. Historically in China, the endemic city of leprosy was much higher along the coast and in the Yangtze Valley. Based on the definition of WHO for elimination of leprosy as a public health problem (a prevalence of less than 1 case per 10,000 residents), China had eliminated leprosy at the national level in 1981 and at the provincial level in 1992 [4]. The Chinese Ministry of Health (MOH, now the National Health Commission), published a national leprosy control plan (2011–2020) in 2011, aiming at controlling leprosy and its harms through public health investment directly allocated for leprosy control [5]. LEPMIS is an updated version of the original National Leprosy Recording and Reporting System. Data from LEPMIS indicated that both the prevalence and the case detection rate of leprosy significant declined between 2010 and 2018. The national prevalence was 0.178 per 100,000 and the detection rate was 0.037 per 100,000 residents in 2018. A total number of 6,602 new cases were detected from 2011 to 2018, and 11.5% of them were detected among people migrating from traditionally leprosy endemic areas to major cities [1].

Zhejiang Province is located in the south of the Yangtze River Delta along the southeastern coast of China. This coastal province is divided into 11 municipalities and subdivided into 93 counties/districts. Zhejiang Provincial Institute for Dermatoses Prevention and Cure is in charge of leprosy control of the whole province. Zhejiang is one of the smallest provinces in China with a population of 57.37 million and a land area of 105,400 square kilometers, about 1% of China. More than 16 thousand cases have been registered since 1949. The highest average detection rate was 2.79 per 100,000 residents in the year 1955–1959, and the highest 5-year average prevalence was 26.75 per 100,000 residents in the year 1970–1975. Zhejiang was one of the leprosy-endemic provinces in China. MDT recommended by WHO was implemented in 1980s and the goal of leprosy elimination by Chinese level (less than 1 per 100,000) was achieved in 1995, and it is the fourth province reaching this goal in China. Since then, case-finding modes of leprosy cases has changed from being active to passive. In order to explore the early detection of new cases under the low epidemic condition, reduce the incidence of G2D deformity and eliminate the harm of leprosy, Health Commission of Zhejiang Province established a monitoring system for suspicious symptoms of leprosy in the whole province in September 2011, which was implemented officially on January 1, 2012. Cases of suspicious symptoms of leprosy found in all medical institutions must be referred to the designated institution for further diagnosis, and the 16 designated institution are located in 11 cities in Zhejiang Province. For each confirmed case of leprosy, the first person who reports the possibility of leprosy is provided a reward of 1,000 RMB, and the doctor who diagnoses a patient is also provided a reward of 1,000 RMB by Zhejiang Provincial Department of Finance. The implementation of suspicious monitoring system of leprosy is valuable for early detection and helps to decrease the disability rate which is suitable in low epidemic areas [6–7].

In Zhejiang Province, the first leprosy case of floating population from other province in China was reported In 1995 and it became an increasing trend of the proportion of floating population among newly detected cases in 2011–2019. In order to understand the temporal-spatial distribution characteristics of newly detected leprosy cases, and provide the scientific rationales for the development of leprosy control strategy, time, space and population distributions were described.

## Materials and methods

### Ethical statement

This study was approved by the Ethical Committee of Zhejiang Provincial Institute for Dermatoses Prevention and Cure. The approval number Is LL2018-03. An informed consent was signed, all the data was anonymized and the patients’ confidentiality was strictly respected in the data processing and analysis.

### Diagnosis and classification of leprosy

Diagnosis of leprosy was based on clinical, bacteriological, and histopathological profiles. All the suspicious leprosy cases in Zhejiang Province were asked to be transferred to the designated institution for confirmation. Ridley-Jopling classification was used to classify the clinical types into I, TT, BT, BB, BL or LL. Two-group classification system developed by WHO was used, and cases were classified into multibacillary (MB) type or paucibacillary (PB) type. The newly detected cases were the cases diagnosed with leprosy, including that moved from other provinces to Zhejiang after diagnosis in that year. Local residents cases were the cases detected among residents of Zhejiang Province, while the floating population cases (foreign born leprosy cases) were the cases detected among residents out of Zhejiang Province. Most of the floating population came to Zhejiang province to make a living, and most of them lived in the same county/district. They often went back to their hometown before the Spring Festival, and back to Zhejiang after the Spring Festival. The early case was a newly detected case with no abnormal deformity or irreversible neurological damage within 2 years course of the disease.

Geographic information system software—ArcGIS 10.4 was used to draw the statistical maps and Geoda 1.14.0 for local spatial autocorrelation analysis and calculating the Getis coefficient. Spatial data processing software—SaTScan 9.5 was used for space scanning statistics of the years.

### Study population

This descriptive retrospective study was based on all the newly detected leprosy cases in 2011–2019 in Zhejiang Province.

### Data collection and statistical analysis

Data on the demographic of Zhejiang Province from 2011 to 2019 were obtained from the China Information System for Disease Control and Prevention, and the epidemiological data on newly detected leprosy cases were obtained from the LEPMIS.

Excel 2007 was used to compile the data of newly detected leprosy cases, calculated the age of the cases according to the birth date and the diagnosis date, and described the basic demographic characteristics, time distribution trend and regional distribution characteristics of the cases according to the ID number, gender, age, occupation, incidence date, diagnosis date and other basic information of the cases. All the data was put into software Statistical Package for Social Sciences (SPSS) version 23.0. Qualitative variables were presented as frequencies and percentages, and quantitative variables were presented as the mean±standard deviation. The Chisquare Test was used to compare categorical variables, and T-test was used to compare numerical variables. The geographic information system software—ArcGIS 10.4 was used to draw the statistical maps, and Geoda 1.14.0 was for local spatial autocorrelation analysis (local Getis coefficient method). Spatial autocorrelation refers to the correlation of a variable on the adjacent space position‚ which concentrates of a metric space unit attribute value. It is aimed to discover the spatial distribution characteristics of a particular region. In this study, the local Getis coefficient method was used to explore the aggregation region, which showed the hot areas and the cold areas [8]. P-value less than 0.05 was considered significant.

## Results

### Demographic information

A total of 167 newly detected leprosy cases were reported in Zhejiang Province during 2011–2019. Among the 167 patients, there were 107 males and 60 females, with a male to female ratio of 1.78. The male to female ratio of floating population cases (2.37:1, 90/38) was higher than that of local residents cases (0.77:1, 17/22), and the difference was statistically significant (*X*^2^ = 9.273, *P* = *0*.*002*). The mean age at diagnosis was 37.99±14.81 years, ranging from 10 to 84 years. Two children cases (less than 15 years old) were notified in this period (Table 1). Distribution of the occupation was workers (81, 48.50%), farmers (49, 29.34%), household and unemployed (18, 10.78%), business services (4, 2.40%), retirees (4, 2.40%), students (3, 1.80%), individuals (2, 1.20%), catering services (1, 0.60%), other occupations (5, 2.99%).

**Table 1:** Population distribution of newly detected leprosy cases in Zhejiang, 2011–2019.

Year	Newly detected cases	Male	Female	Average age (years)	Local residents cases		Floating population cases		T-test of average age	
					Number of cases	Average age (years)	Number of cases	Average age (years)	*T* value	*P value*
2011	31	18	13	38.87±14.61	7	61.00±12.37	24	32.42±6.78	8.053	<0.001
2012	24	16	8	37.83±15.86	4	56.25±20.12	20	34.15±12.44	2.936	0.008
2013	19	14	5	33.84±11.03	3	47.00±17.52	16	31.38±7.80	2.581	0.019
2014	25	18	7	39.32±16.68	6	61.33±16.50	19	32.37±9.10	5.555	<0.001
2015	18	11	7	44.5±15.14	8	52.38±18.57	10	38.20±8.11	2.180	0.045
2016	13	10	3	31.62±13.21	2	46.50±27.58	11	28.91±8.99	1.916	0.082
2017	15	7	8	43.47±16.64	5	51.60±19.39	10	39.40±14.45	1.381	0.191
2018	11	5	6	34.27±8.98	2	28.50±0.71	9	35.56±10.03	0.954	0.365
2019	11	8	3	31.18±12.42	2	44.00±21.21	9	28.33±9.29	1.781	0.109
**Total**	**167**	**107**	**60**	**37.99±14.81**	**39**	**53.23±17.55**	**128**	**33.23±10.00**	**9.067**	**<0.001**

### Epidemiological information

There were 157 (94.01%) cases classified as MB type and 10 (5.99%) cases as PB patients. The most common detection mode was through skin-clinics (157, 94.01%), followed successively by referral by other departments of general hospitals (3, 1.80%), self-report (3, 1.80%), on-site survey (2, 1.20%), report by others (1, 0.60%) and contact examination (1, 0.60%) (Table 2). There were 24 cases with the transmission way of family, 1 was local residents case, and 23 were floating population cases.

**Table 2:** Epidemiological data of newly detected leprosy cases in Zhejiang, 2011–2019.

Year	Newly detected cases	Case detection rate (1/100,000)	Cases of MB (%)	Average delay time (months)	Cases with G2D	Proportion of G2D (%)	Number of cases detected by skin-clinics	Proportion of skin-clinics detection (%)
2011	31	0.06	29 (93.55%)	40±35	7	22.58	30	96.77
2012	24	0.04	22 (91.67%)	23±19	4	16.67	19	79.17
2013	19	0.03	17 (89.47%)	18±15	3	15.79	18	94.74
2014	25	0.05	25 (100.00%)	16±14	1	4.00	24	96.00
2015	18	0.03	17 (94.44%)	17±19	3	16.67	17	94.44
2016	13	0.02	13 (100.00%)	12±7	1	7.69	13	100.00
2017	15	0.03	14 (93.33%)	15±12	1	6.67	15	100.00
2018	11	0.02	11 (100.00%)	16±10	3	27.27	10	90.91
2019	11	0.02	9 (81.82%)	11±16	1	9.09	11	100.00
**Total**	**167**	**0.03**	**157 (94.01%)**	**21±22**	**24**	**14.37**	**157**	**94.01**

### Delay in diagnosis and G2D

The average interval of patient delay from the onset of the disease to the establishment of diagnosis was calculated in months. The shortest delay time of the 167 newly detected leprosy cases was 1 month, and the longest delay time was 150 months in 2011. The latter case was from Sichuan Province (a highly endemic province in China). The average delay time from 2011 to 2019 was 21 months, with the shortest 11 months in 2019 and the longest 40 months in 2011. The proportion of newly detected leprosy cases in early detection increased year by year, with the lowest in 2011 (45.16%, 14/31) and the highest in 2019 (90.91%, 10/11), and the average proportion in these 9 years was 69.46% (116/167).14.37% (24/167) of the newly detected leprosy cases presented with G2D at diagnosis. The annual G2D proportion showed an obvious declining trend in this period, from 22.58% in 2011 to 9.09% in 2019 (Table 2). The average delay time of G2D cases were significantly longer (39 months) than that (18 months) of cases without G2D. Among the 24 cases with G2D, the proportions of cases with patient delay of >12 months (20/24, 83.33%), delay time of >24 months (17/24, 70.83%).

Three of the 11 newly detected leprosy cases in Zhejiang Province in 2018 were with G2D, and two of them were diagnosed in other provinces in China, coming to Zhejiang for further treatment. In 2018, the G2D proportion was 27.27%. If not considering these two cases that came to Zhejiang after they were diagnosed, the adjusted G2D proportion was 11.11% (1/9).

### Temporal distribution of newly detected cases

The annual number of newly detected leprosy cases showed a decreasing trend from 2011 to 2019 (Table 1), ranging from 31 in 2011 to 11 in 2019. From 2016 to 2019, the number of annual leprosy cases was rather stagnant. A drastic decline occurred between 2014 and 2016. From 2016, the annual number of cases reached a lower plateau.

### Spatial distribution of newly detected cases

**Fig 1:**
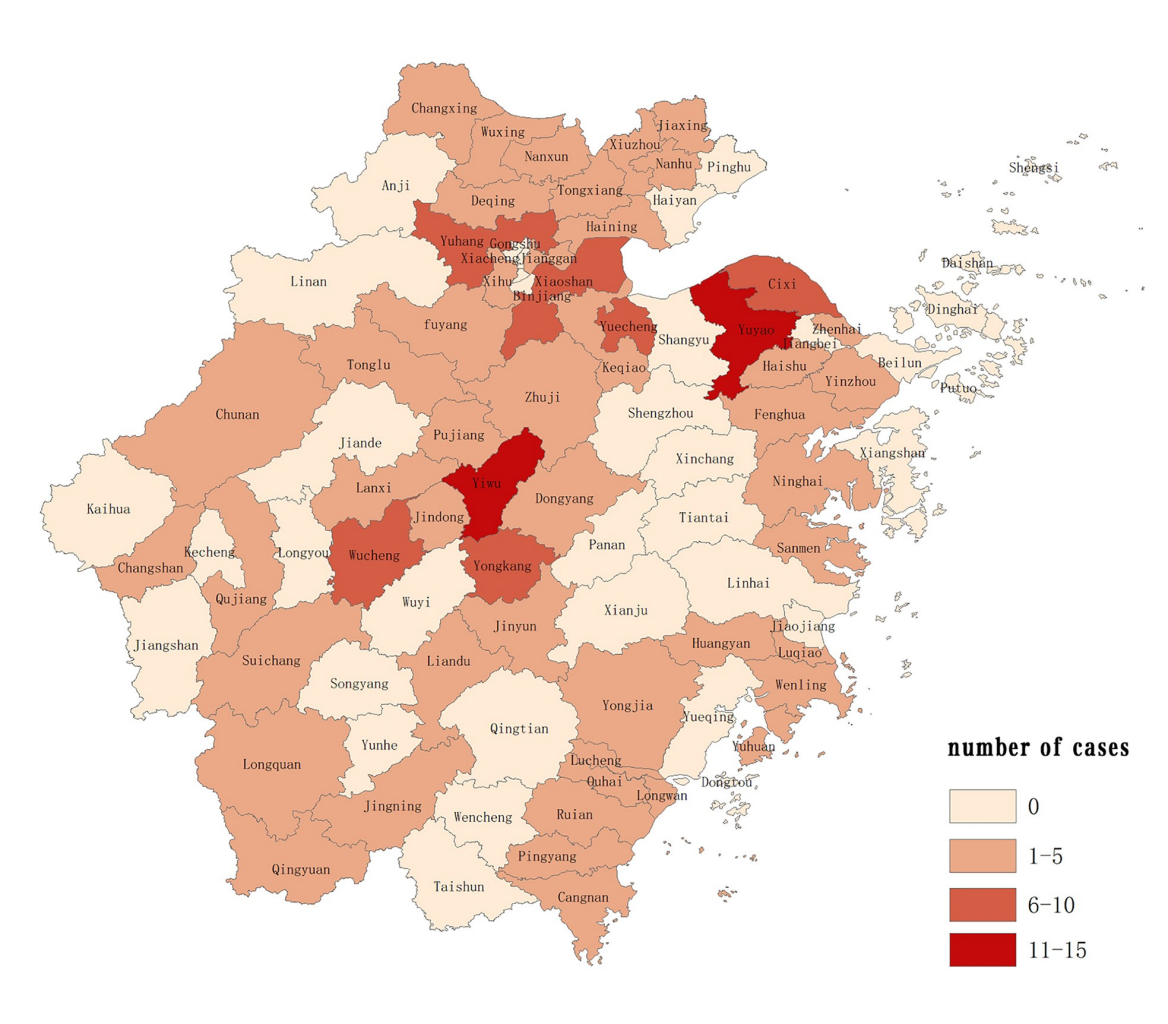
Geographical distribution of newly detected leprosy cases in Zhejiang Province, 2011–2019.

During this study period, 167 newly detected leprosy cases were scattered in 10 (90.91%) municipalities, 55 (59.14%) counties/districts, and the distribution was unbalanced. At the municipal level, there were 39 cases, 31 cases, 22 cases and 20 cases in the 4 municipalities: Jinhua, Ningbo, Hangzhou and Wenzhou respectively. At the county level, the number of newly detected leprosy cases was less than 10, except Yiwu (14 cases) and Yuyao (11 cases) (Fig 1).

Out of the 167 cases, 39 (23.35%) were born in Zhejiang Province and 128 (76.65%) were floating population (from other 12 provinces in China). The total number of cases of the 12 provinces ranged from 1 to 73. Most of the floating population cases came from Guizhou Province, Yunnan Province and Sichuan Province, which are high endemic provinces in southwestern part of China. Newly detected cases were detected every year in these 3 provinces, but there is no significant growing or slowing trend of cases from some provinces only one or two during 2011–2019 (Table 3). **I**n those 128 floating population cases, 38 were female and 90 were male. The proportion of floating population in male was significantly higher than that in female (*P*<0.001). The mean age of floating population cases was 28.33±9.29 years, which is significantly younger than that of native cases 53.23±17.55 years, P<0.001) (Table 1).

**Table 3:** Distribution of 167 newly detected leprosy cases based on the registered provinces, 2011–2019.

Province	2011	2012	2013	2014	2015	2016	2017	2018	2019	Total	proportion (%)
Guizhou	15	9	11	13	4	5	6	5	5	73	43.71
Zhejiang	7	4	3	6	8	2	5	2	2	39	23.35
Yunnan	3	4	2	1	3	2	1	2	2	20	11.98
Sichuan	4	1	1	2	2	2	2	1	1	16	9.58
Hunan	1	2	0	1	0	1	0	0	0	5	2.99
Jiangxi	1	0	0	0	1	0	0	0	1	3	1.80
Shanxi	0	0	0	1	0	1	0	0	0	2	1.20
Chongqing	0	1	0	1	0	0	0	0	0	2	1.20
Henan	0	1	1	0	0	0	0	0	0	2	1.20
Anhui	0	0	1	0	0	0	0	1	0	2	1.20
Guangxi	0	1	0	0	0	0	0	0	0	1	0.60
Hubei	0	1	0	0	0	0	0	0	0	1	0.60
Liaoning	0	0	0	0	0	0	1	0	0	1	0.60
**Total**	**31**	**24**	**19**	**25**	**18**	**13**	**15**	**11**	**11**	**167**	**100.00**

**Fig 2:**
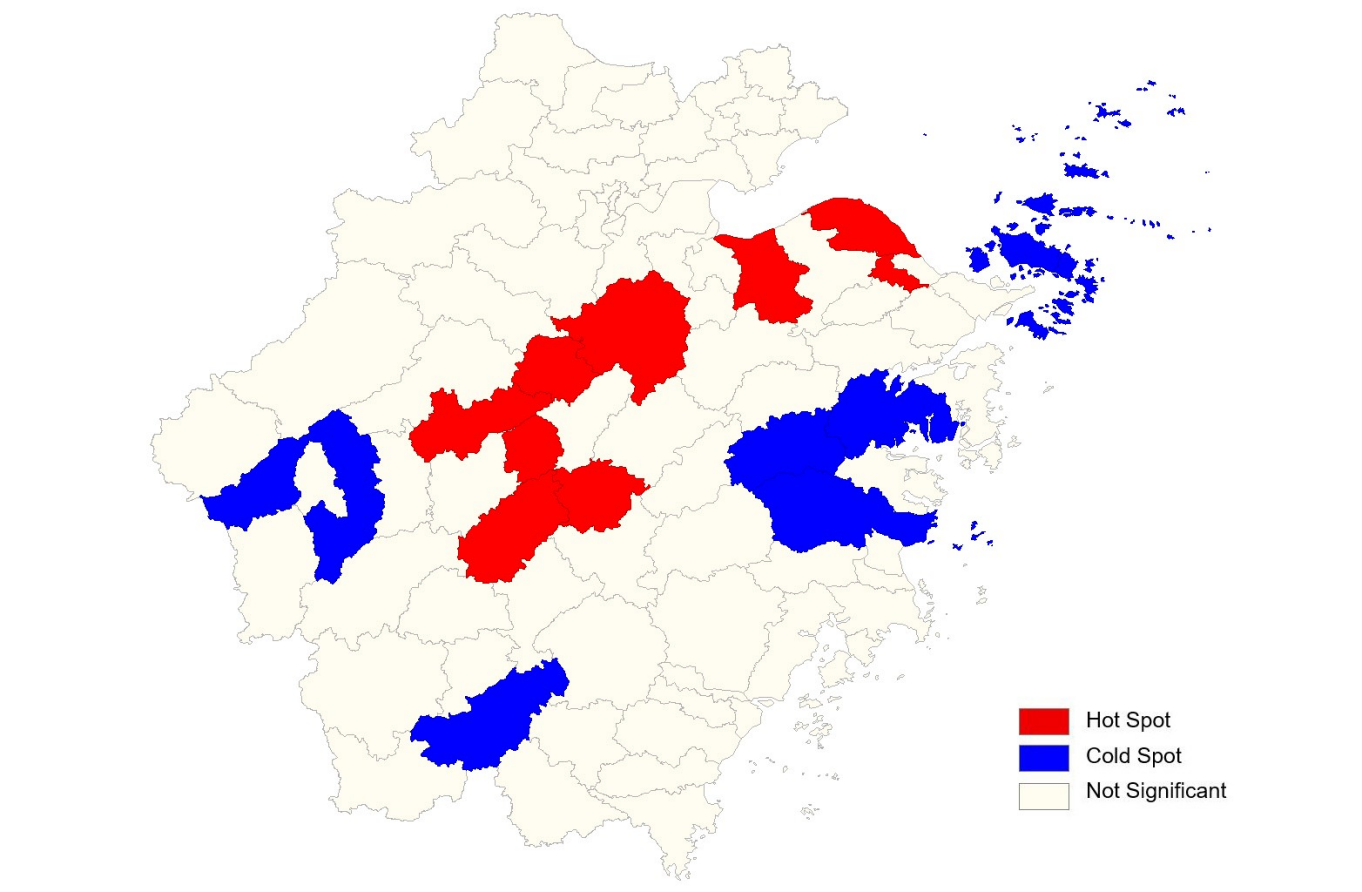
Local spatial autocorrelation analysis of newly detected leprosy cases in Zhejiang Province, 2011–2019. Different colors represent the types of spatial autocorrelation between different regions. The red represents the statistically significant of high-high positive correlation region, that is, the counties/districts were with a high incidence, and the adjacent counties/districts were also with a high incidence.

The results of local indicators of autocorrelation (LISA) analysis showed that the high-high areas (Hot Spot) were mainly concentrated in the middle and northeast of Zhejiang Province, while the low-low areas (Cold Spot) were concentrated in the east and southwest (Fig 2).

## Discussion

Data from LEPMIS indicated that from 2011 to 2019, the detection rate of annual newly detected leprosy cases in Zhejiang Province was generally in a low epidemic state and showed a declining trend of new cases detection. A total of 2,479 newly detected leprosy cases were reported in 2018 in China, and the detection rate of new cases in Zhejiang Province (0.02/100,000) was much lower than that of China (0.037/100,000) and the global average (2.74/100,000) [1]. The average delay time of newly detected leprosy cases became shorter, from 40 months in 2011 to 11 months in 2019, the annual G2D proportion showed an obvious declining trend in this period, from 22.58% in 2011 to 9.09% in 2019, significantly lower than that of other provinces in China [9–10]. The proportion in early detection increased year by year, from 45.16% in 2011 to 90.91% in 2019. That was due to the monitoring system for suspicious symptoms of leprosy in Zhejiang Province, which was established implemented officially by Health Commission of Zhejiang Province on January 1, 2012. The implementation of suspicious monitoring system of leprosy is valuable for early detection and helps to decrease the disability rate, especially in post-elimination era and in low endemic areas [11]. The average delay time of G2D cases were significantly longer (39 months) than that (18 months) of cases without G2D. In order to achieve the Global Leprosy Strategy 2016–2020 target of 0 new pediatric cases with G2D by 2020 [12], efforts are still needed to strengthen the training of dermatologists in medical institutions considering that 94.01% of the newly detected leprosy cases were detected by skin-clinics, and the relationship between delay time and G2D in post-elimination era.

Newly detected leprosy cases originated from floating population is a challenge for leprosy prevention and control in Zhejiang Province. During the period of 2011–2018 in China,11.5% of new cases were detected among people migrating from traditionally leprosy endemic areas to major cities such as Beijing, Shanghai, Guangzhou, and Shenzhen [1]. Most of the floating population cases (109/128, 85.16%) came from Guizhou Province, Yunnan Province and Sichuan Province, which are the less developed provinces in Chinese. Cases from these provinces came to work in Zhejiang Province in order to make a living. There was no associated local and floating cases found in the same geographical area and same time period. Since there were 23 floating population cases with family history, it was necessary for us to carry out an inter-provincial epidemiological collaboration to follow the cases and their contacts in the province from they came and return to Zhejiang. The mean age at diagnosis of Local residents cases was about 53 years old, which was one of the signs of the late epidemic stage of leprosy and was close to the epidemic level in developed countries. While the mean age of the floating population cases was about 33 years old, which was significantly younger than that of the local residents cases. It indicated that the epidemic situation in the endemic provinces in the southwestern of China was still not optimistic. Meanwhile, the monitoring of the floating population from provinces with high prevalence of leprosy should be strengthened in order to detect the cases in the early stage. The results of the study showed that 94.01% of the newly detected cases were detected by skin-clinics. It prompted that training of dermatologists in medical institutions should be strengthened as an important means of leprosy cases detection.

In this study, the spatial distribution of the 167 newly detected leprosy cases showed that the distribution is uneven and scattered at the municipal or the county level. 76.65% of the 167 cases were from other 12 provinces in China, and most of them came from the high endemic provinces in China. That is not like other communicable diseases and non-communicable diseases in Zhejiang Province. The proportion was much higher than that of China and most of the Member States and territories of World Health Organization [3,13]. It is predicted that in the next decade, the newly detected cases in Zhejiang Province will still mainly from floating population with the development of economy and the migration. This suggests that we should strengthen inter-provincial cooperation between low-prevalence and high-prevalence areas to reduce the incidence of leprosy. Among the 167 cases, two children cases, without G2D, were detected in this period and came from Guizhou Province (one of the high epidemic provinces in China) with a family history. This indicated that efforts are still necessarily needed to strengthen the early detection and standard management of the leprosy cases in floating population, and we should pay more attention to the second-generation cases of leprosy in the floating population. Contact tracing- examination of persons having contact with a leprosy patient is a very important component to identify populations at high risk for developing leprosy [14]. Providing chemoprophylaxis may be also one of the effective ways to prevent contacts of leprosy patients from contracting the disease [15–16]. At the same time, we should also do a good job in health education to prevent the transmit of leprosy.

The results of local indicators of autocorrelation (LISA) analysis showed that the high-high areas were mainly concentrated in the middle and northeast of Zhejiang Province. These areas are economically developed areas with a large proportion of the floating population, where the floating population cases can get more employment opportunities, and the cost of living was not too high. In order to achieve the targets of China National Program for Eliminating Harms Due to Leprosy (2010−2020) and WHO Global Leprosy Strategy 2016−2020: Accelerating towards a leprosy-free world, prevention and control work should be strengthened in these areas. Data from existing databases of our manuscript shows that the new cases were not clustered in the shared boundaries. The low-low areas (Cold Spot) were concentrated in islands to the east and the mountains to the southwest, and this may have something to do with the small number of floating population in these places.

## Conclusion

A few scattered cases still can be seen in post-elimination era. The implementation of suspicious monitoring system of leprosy is valuable for early detection which is suitable in low leprosy epidemic areas. There was a spatial clustering of the newly detected leprosy cases, the high-high areas were mainly concentrated in the middle and northeast of Zhejiang Province in Zhejiang Province, and effective strategies are needed to strengthen leprosy control in these areas. Most of the newly detected leprosy cases in Zhejiang Province were from other high epidemic Province in China. It has brought a new challenge for leprosy control and prevention in post- elimination era in Zhejiang. It is necessary to carry out an inter-provincial epidemiological collaboration to follow the cases, and strengthen the early detection and standard management of the leprosy cases in floating population in Zhejiang.

### Limitations

There is still a lack of effective evidence to determine whether the floating population cases were infected before or after coming to Zhejiang Province. It remains to be further confirmed by case tracing and genetic testing, and that is the study we're carrying on now.
